# Prevalence of low back pain by anatomic location and intensity in an occupational population

**DOI:** 10.1186/1471-2474-15-283

**Published:** 2014-08-21

**Authors:** Matthew S Thiese, Kurt T Hegmann, Eric M Wood, Arun Garg, J Steven Moore, Jay Kapellusch, James Foster, Ulrike Ott

**Affiliations:** Department of Family and Preventive Medicine, Rocky Mountain Center for Occupational & Environment Health, School of Medicine, University of Utah, 391 Chipeta Way, Suite C, Salt Lake City, UT 84108 USA; Center for Ergonomics, University of Wisconsin-Milwaukee, P.O. Box 784, Milwaukee, WI 53201 USA; Occupational Science & Technology Department, College of Health Sciences, University of Wisconsin – Milwaukee, PO Box 415, Milwaukee, WI 53201 USA; School of Rural Public Health, Texas A&M, University Health Science Center, College Station, TX 77843-1266 USA

**Keywords:** Low back pain, Point prevalence, 1-month period prevalence, Intensity, Location, Epidemiological, Cross-sectional analysis

## Abstract

**Background:**

Low Back Pain (LBP) is a common and costly problem, with variation in prevalence. Epidemiological reports of rating of pain intensity and location within the low back area are rare. The objective is to describe LBP in a large, multi-center, occupational cohort detailing both point and 1-month period prevalence of LBP by location and intensity measures at baseline.

**Methods:**

In this cross-sectional report from a prospective cohort study, 828 participants were workers enrolled from 30 facilities performing a variety of manual material handling tasks. All participants underwent a structured interview detailing pain rating and location. Symptoms in the lower extremities, demographic and other data were collected. Body mass indices were measured. Outcomes are pain rating (0–10) in five defined lumbar back areas (i) LBP in the past month and (ii) LBP on the day of enrollment. Pain ratings were reported on a 0–10 scale and subsequently collapsed with ratings of 1–3, 4–6 and 7–10 classified as low, medium and high respectively.

**Results:**

172 (20.8%) and 364 (44.0%) of the 828 participants reported pain on the day of enrollment or within the past month, respectively. The most common area of LBP was in the immediate paraspinal area with 130 (75.6%) participants with point prevalence LBP and 278 (77.4%) with 1-month period prevalence reported having LBP in the immediate paraspinal area. Among those 364 reporting 1-month period prevalence pain, ratings varied widely with 116 (31.9%) reporting ratings classified as low, 170 (46.7%) medium and 78 (21.4%) providing high pain ratings in any location. Among the 278 reporting 1-month period prevalence pain in the immediate paraspinal area, 89 (32.0%) reported ratings classified as low, 129 (46.4%), medium and 60 (21.6%) high pain ratings.

**Conclusions:**

Pain ratings varied widely, however less variability was seen in pain location, with immediate paraspinal region being the most common. Variations may suggest different etiological factors related to LBP. Aggregation of different locations of pain or different intensities of pain into one binary classification of LBP may result in loss of information which may potentially be useful in prevention or treatment of LBP.

**Electronic supplementary material:**

The online version of this article (doi:10.1186/1471-2474-15-283) contains supplementary material, which is available to authorized users.

## Background

Lifetime prevalence of low back pain (LBP) is reportedly 75-84% of the general population studied in developed countries, which includes working individuals, but also includes individuals who disabled and are not employed[1–6]. Direct costs for LBP are estimated between $20 billion and $98 billion in the US, with indirect annual costs included cost estimates are as high as $200 billion[7, 8]. The point prevalence of LBP among reported epidemiological studies of the general population in developed countries including Canada and the US ranges from 4.4% to 33.0% and 1-month period prevalence has ranged from 35% to 52.2%[2, 5, 8–15].

LBP prevalence is a common reported outcome, yet it is rarely well defined and reproducible[1–6, 8–16]. When epidemiological and clinical studies do define LBP, it has been generally defined as pain between the 12^th^ rib and the gluteal fold[2, 5, 8, 9, 11]. We could not identify a single study that subdivided this area in order to more adequately describe LBP. Additionally, many studies assess pain rating, but simply aggregate all pain together[2, 5, 8–15]. While many randomized trials report mean pain ratings in the low back area, only two population based epidemiological articles described pain intensity in the low back area, but none could be identified that differentiated different regions of pain in the lumbar spine with stratified pain intensities[12, 17]. There is little reported regarding the pain rating or location distribution in a non-clinical population.

Limitations in LBP research include: i) subjective and inconsistent definitions of LBP regarding location and pain intensity[18–22], ii) limited use of objective outcome measures as compared to the occurrence of purely subjective outcomes[19–21, 23], iii) infrequent reporting of multiple LBP prevalence measures from the same population to allow for comparability[11, 20, 21, 23, 24], iv) lack of detailed LBP anatomical location descriptions[20–22], and v) rarely reported pain ratings[20, 22, 24]. Conflicting results in the epidemiological literature have led to a recent consensus statement that recommended inclusion of intensity and duration measures in case definitions of LBP[22]. Still, there are no epidemiological reports detailing the anatomical location and pain intensities of low back pain in a large population. A review of 906 Randomized Controlled Trials for treatment of LBP identified that back pain is typically defined as any pain between the thorax and gluteal fold, encompassing a wide range, yet without definition to ascertain whether patterns of pain may denote different subgroups that are either more prone toward chronicity and/or responsive to different treatment options[25].

The objectives of this report are to describe by anatomic location the: (i) point and 1-month prevalence of LBP, and (ii) intensity of LBP in a large, multi-center, multi-plant occupational cohort. Additional analyses include assessment of lifetime prevalence of LBP, prevalence of symptoms into the lower extremities, and descriptions of lower extremity symptoms by location. This descriptive study is the first in a series of articles exploring these relationships in detail.

## Methods

The Institutional Review Boards of the University of Utah, University of Wisconsin-Milwaukee and Texas A&M University approved this study. All participants provided written informed consent prior to enrolling in this study. This study conforms to the Strengthening and Reporting of Observational studies in Epidemiology (STROBE) recommendations. The study protocol has been described in detail elsewhere, thus a brief description of the methods is below[26]. The cohort data were collected from 2003–2012, with baseline data collected between 2003 and 2006.Participants were enrolled from 27 employers with 30 diverse facilities located in Illinois, Texas, Utah and Wisconsin, USA. Study participants employed at these facilities performed a variety of operations, including (a) poultry and meat processing; (b) baking; (c) printing; (d) order selection in grocery warehousing; (e) book packaging, clothing packaging, and palletizing in a distribution center; and (f) manufacturing of cabinets, glass windows and doors, electric lights, chemicals, garage doors, lawnmowers, airbags and automotive parts, small engines, metal parts, plastic parts, office chairs, ice cream, salt, and cosmetics. Production managers were enrolled if they were involved in manual material handing tasks that could be quantified. Workers with unpredictable job physical exposures if quantification of physical exposures would be difficult, including supervisors, fork lift operators, and clerical workers were not invited to participate.

Participating employers were approached because a majority of their employees performed manual material handling tasks, and thus the study population represents these workers, but is also similar in demographic measures to other working populations in developed countries, including another cohort by the same study group[27–32]. Participants were invited based on estimates made by research team members of the Revised NIOSH Lifting Equation (NIOSH RLE) and were classified equally into low, medium and high rough exposure groups after performing preliminary analyses of a sample of jobs, blinded to health status of workers. The NIOSH RLE has been applied to estimate association between LBP and job physical factors. Participants were enrolled regardless of whether they did or did not have low back pain[33]. The cohort was created to evaluate relationships between a broad spectrum of job physical factors, including those used by the NIOSH RLE and the development of LBP. Eligible participants were: (i) at least 18 years of age, (ii) able to give informed consent, and (iii) without plans to retire or leave their employer within four years. Workers with probable frequent and unpredictable changes in job physical exposures were excluded. Multiple enrollments were performed at most plants to increase participation and reduce selection bias. Exact participation rates are not known, however estimated participation is greater than 75% at each individual plant, and as high as 96% at an individual plant. All workers at each plant who met the eligibility requirements were invited to participate, with an overall participation of >75% of all eligible workers. 897 were consented; however some (n = 69) did not have complete baseline data and were excluded from these analyses. All participants were employed and performing their normal job duties. Data collection occurred during normal work hours for these participants, and included baseline health and job measures beginning in 2003, monthly health assessments, and quarterly job assessments for changes. All enrollments and monthly follow ups were conducted at the participating company, however all data collected were kept strictly confidential. All efforts were taken to ensure privacy and confidentiality throughout the data collection process. All of the individuals involved in data collection, storage, analysis or dissemination of results were part of the research team and not employed by any of the companies involved in the study.

The Health Outcomes Assessment Team administered computerized questionnaires and computerized structured interviews after consent was obtained. These data instruments were developed for this study based on many reported associations or suspected risk factors relating to LBP[34]. These questionnaires and structured interview questions were constructed from prior epidemiological studies[35–37]. All data collection instruments were extensively pre-pilot, pilot, and field tested to ensure accuracy and reproducibility prior to data collection. The questionnaire included medical health items and psychosocial factors including: (a) demographics, (b) past medical history, (c) psychosocial questions, and (d) other questions (e.g., sleeping patterns, smoking, and alcohol consumption). The questionnaire and structured interview questions are available in the study protocol paper[26]. Monthly assessments included focused questions about past pain, inquiries regarding new pain, and a focused physical exam if warranted. The monthly questions querying location and quality of LBP were the same as those for baseline.

**Figure 1:**
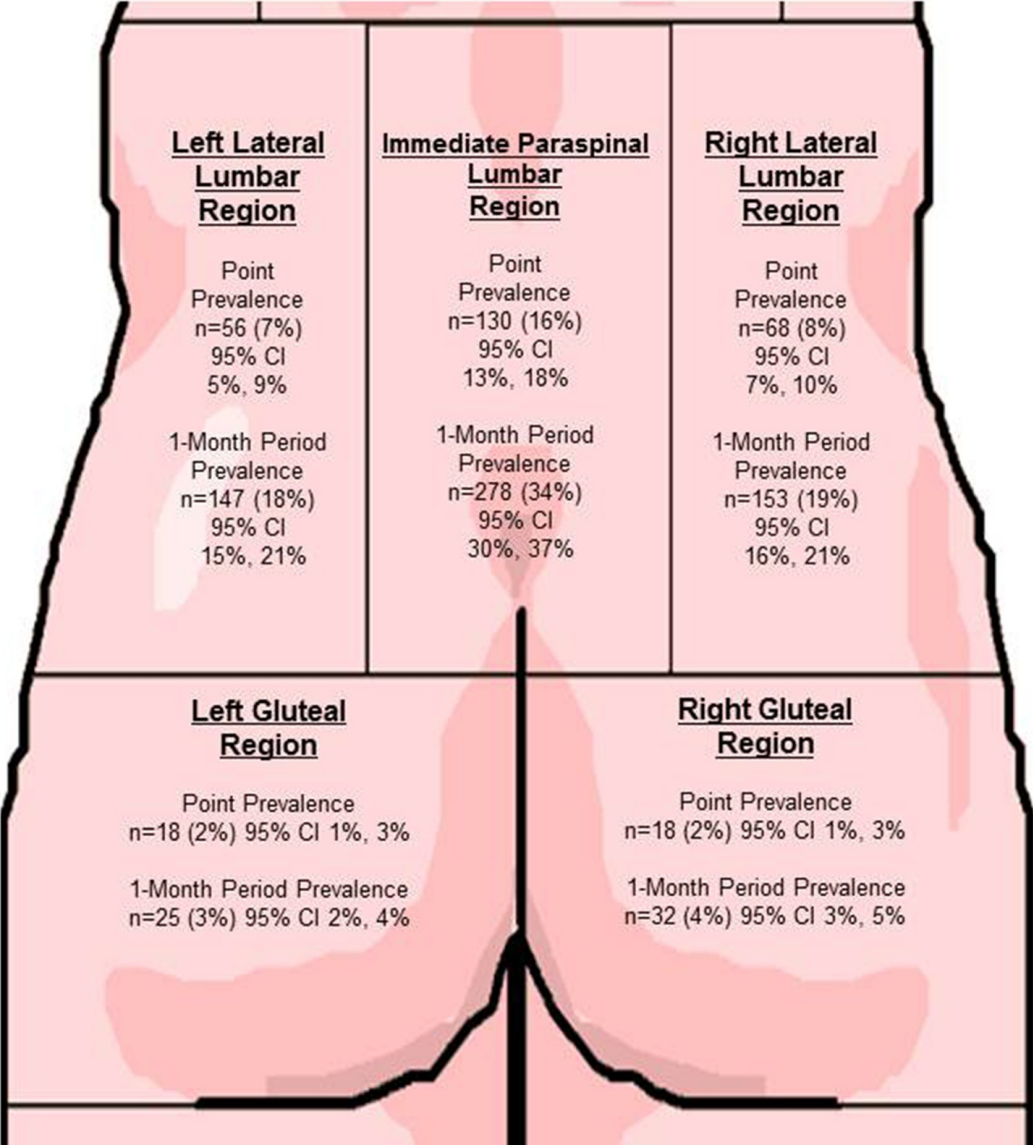
**Point prevalence and 1-month period prevalence of low back pain overlaid on the body diagram used with workers for anatomical localization of pain from a large (n = 828) multicenter occupational cohort in the US.**

The computerized structured interview was administered by trained Physical Therapists or Occupational Medicine Residents. Interviewers were standardized annually and followed the computerized questionnaire format, asking the same questions in the same order. LBP, pain ratings on a 0–10 scale, and pain anatomical location(s) were collected separately for each timeframe of: i) the day of enrollment, and ii) in the past month[26]. Tingling and/or numbness in the lower extremities in the past month were also collected; these two symptoms were not differentiated. Pain location was collected by the interviewer using a standardized body diagram (See Figure 1) and subsequent questions to determine the exact location and extent of the pain. A pain drawing was completed by the participant to assist in the identification of pain in the low back, specifically pain in multiple locations, however specific information regarding pain in each of the areas noted in Figure 1 were collected by the interviewer. Symptoms were recorded for all low back areas identified with having had pain any time in the past month. These divisions on the standardized body diagram were defined *a priori* by the research team. Height and weight were measured to calculate body mass indices (BMI).

### Case definitions

Case definitions for this report, each with 0–10 pain ratings, include: (i) LBP in the past month and (ii) LBP on the day of enrollment. Pain ratings of 1–3, 4–6, and 7–10 out of 10 were categorized into low, medium and high pain categories, respectively[38, 39]. Pain anatomical locations were categorized into one or more of 5 lumbar areas: (i) Left Lateral Lumbar area, (ii) Lumbar immediate paraspinal area, (iii) Right Lateral Lumbar area, (iv) Left Gluteal area, and (v) Right Gluteal area (Figure 1).

### Statistical analysis

All analyses were conducted using SAS 9.2 (SAS Institute, Cary NC). Given the goal of this study is to report the prevalence of LBP, data are presented with descriptive statistics including mean ± standard deviation, frequency, percent, and 95% confidence interval around prevalence estimates.

## Results

There were 828 study subjects with complete baseline health data who were included in these analyses (see Table 1). Most of the subjects were male (n = 529, 63.9%), with a mean age of 38.8 ± 12.0 years and BMI of 29.3 ± 6.5 kg/m[2]. Slightly more than half were never smokers (n = 458, 55.3%) and 192 (23.2%) were past smokers. Few had diagnoses of diabetes mellitus (n = 37, 4.5%), high cholesterol (n = 155, 18.7%) or high blood pressure (n = 119, 14.4%). Few participants (n = 64, 7.7%) had ever been involved in a Workers’ Compensation claim for LBP. Some participants had seen a physician for their LBP (n = 198, 23.9%), had a narcotic prescription for LBP (n = 74, 8.9%) or been diagnosed with sciatica (n = 81, 9.8%). Participants had been working at their current job for a mean of 4.6 ± 5.9 years, and 31 had changed jobs in the past due to LBP.

**Table 1:** **Baseline demographic and health status data from a large (n = 828) multicenter occupational cohort in the US**

	Total population		
**Continuous Data**	**Mean ± SD**	**Median**	**Range**
Age (years)	38.8 ± 12.0	38.1	18.3 – 69.0
Body Mass Index (kg/m^2^)	29.3 ± 6.5	28.3	15.87 – 85.4
Systolic Blood Pressure (mm Hg)	128.8 ± 17.3	128	74 – 235
Diastolic Blood Pressure (mm Hg)	78.3 ± 10.9	78	43 – 147
**Categorical Data**	**Category**	**Frequency (%)**	
Sex	Male	529 (63.9%)	
	Female	299 (36.1%)	
Lifetime Prevalence of LBP	Yes	526 (63.5%)	
Diabetes Mellitus Diagnosis	Yes	37 (4.5%)	
High Cholesterol Diagnosis (>200 mg/dl)	Yes	155 (18.7%)	
High Blood Pressure Diagnosis	Yes	119 (14.4%)	
Tobacco Use	Never	458 (55.3%)	
	Past	192 (23.2%)	
	Current	178 (21.5%)	

### Point prevalence

Approximately one-fifth of the 828 (20.8%) participants reported LBP in any area on the day of enrollment, with the most common location of point prevalent LBP was immediate paraspinal, with 130 (75.6% of those with point prevalent pain) reporting pain in that area (see Figure 1). Of those with baseline or point prevalent pain, 58 (33.7%) reported pain ratings classified as low, 75 (43.6%) medium, and a surprisingly high 39 (22.7%) high pain ratings in any low back area. Similar trends in pain ratings were reported in the immediate paraspinal area, although with lower prevalence. Lateral region LBP pain ratings were similar between the left and right sides. Point prevalent pain was similar, though less common than 1-month period prevalent pain (data not shown).

Among the 172 study participants who reported pain on the day of enrollment, most participants (n = 104, 60.5%) reported pain in only one of the five anatomical regions (See Table 2). However, 68 (39.5%) participants reporting LBP pain on the day of enrollment reported pain in more than 1 area.

**Table 2:** **One-month period and point prevalence – total number of structured body regions reported with pain from a large (n = 828) multicenter occupational cohort in the US**

Regions with pain	Point prevalence frequency, n (%)	Point prevalence cumulative frequency, n (%)	1-month period prevalence frequency, n (%)	1-month period prevalence cumulative frequency, n (%)
No Pain	656 (79.2%)	656 (79.2%)	464 (56.01%)	464 (56.0%)
1 Region	104 (12.6%)	760 (91.8%)	207 (25.0%)	671 (81.0%)
2 Regions	32 (4.0%)	792 (95.7%)	65 (7.9%)	736 (88.9%)
3 Regions	27 (3.3%)	819 (98.9%)	78 (9.4%)	814 (98.3%)
4 Regions	3 (0.4%)	822 (99.3%)	6 (0.7%)	820 (99.0%)
5 Regions	6 (0.7%)	828 (100%)	8 (1.0%)	828 (100%)

### One-month period prevalence

**Figure 2:**
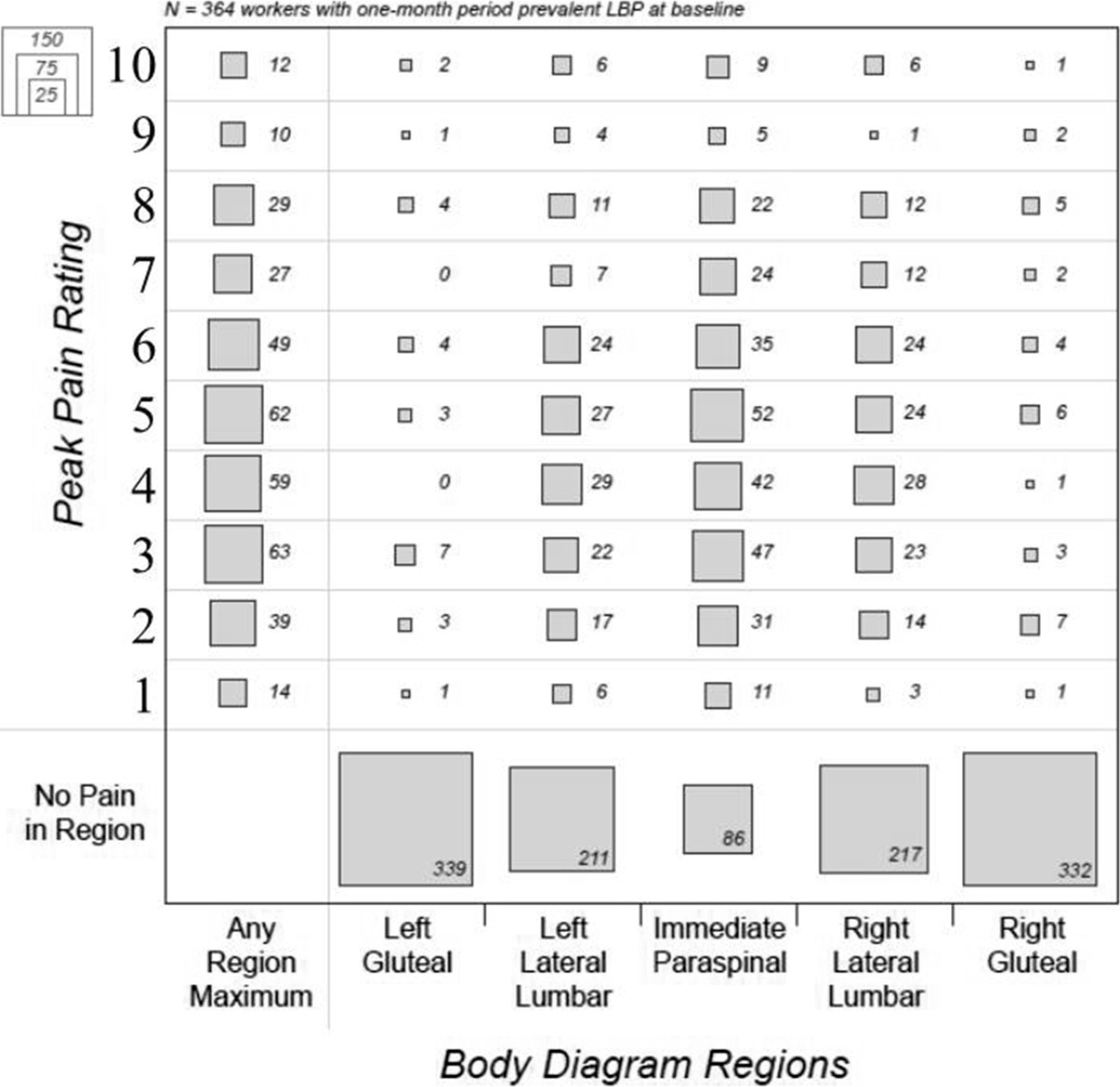
**One-month period prevalence for workers pain severity rating (n = 364) by body diagram region from a large (n = 828) multicenter occupational cohort in the US.** Box size indicates numbers of individuals in the respective pain category (vertical axis) and body diagram region (horizontal axis).

Out of the 828 subjects, nearly half (n = 364, 44.0% of total participants) reported having LBP within the month prior to enrollment. Of those 364 participants, 278 (73.6% of those with 1-month period prevalent pain), reported having pain that was in the immediate paraspinal lumbar area (see Figure 1). Again, the right side had non-significantly higher 1-month prevalence (40.4% and 40.2% respectively).Among those 364 reporting one-month period prevalence LBP, 78 (21.4%) reported pain classified as high in any low back body area (See Figure 2). 60 (16.5%) reporting high pain ratings in the immediate paraspinal lumbar area, with about half as many having reported high pain ratings for each of the lateral lumbar areas. A surprisingly small proportion had pain in the gluteal areas (3-4%). 157 (43.1%) of those reporting 1-month period prevalence reported pain in more than one area.

1-month period prevalence pain location patterns were similar to those of point prevalence; however the pain ratings were consistently higher, regardless of the location or number of areas involved.

### Lower extremity pain, tingling and numbness

Nearly 1/5^th^ of participants reported having ever had LBP that traveled into their legs, however relatively few (n = 59) had those symptoms within the past month (see Table 3).

**Table 3:** **Lifetime prevalence of low back pain traveling into the calf among those who have low back pain from a large (n = 828) multicenter occupational cohort in the US**

Ever traveling into the calf	N = 162 (19.6% of 828)
Right Side	71 (43.8%)
Left Side	54 (33.3%)
Bilateral, at the same time	21 (13.0%)
Bilateral, but at different times	16 (9.9%)

A minority (2.3-2.5%) reported tingling and numbness in any of the lower extremity regions in the prior month (see Table 4). The thigh was the most common location (1.5-1.8%). The frequencies of 1-month period prevalence of tingling and numbness in the right and left lower extremities were comparable on both sides. Approximately 37.5% had tingling and numbness in only one region of one lower extremity with the remainder in more than one lower extremity and/or region (see Table 5).

**Table 4:** **One-month period prevalence of tingling and numbness in the lower extremities from a large (n = 828) multicenter occupational cohort in the US**

	Thigh	Lateral calf	Medial calf	Foot/Feet	Toes	Any region
None	796 (95.1%)	789 (95.3%)	804 (97.1%)	797 (96.3%)	801 (96.7%)	769 (95.1%)
Right Only	15 (1.8%)	15 (1.8%)	9 (1.1%)	12 (1.5%)	11 (1.3%)	24 (2.9%)
Left Only	12 (1.5%)	11 (1.3%)	6 (0.7%)	11 (1.3%)	10 (1.2%)	21 (2.5%)
Bilateral	5 (0.6%)	13 (1.6%)	9 (1.1%)	8 (1.0%)	6 (0.7%)	14 (2.3%)

**Table 5:** **One-month period prevalence of tingling and numbness in the multiple regions (thigh, lateral calf, medial calf, foot/feet, toes) of the lower extremities from a large (n = 828) multicenter occupational cohort in the US**

Number of regions with tingling/numbness	Right leg	Left leg	Both legs
No regions	804 (97.1%)	807 (97.5%)	809 (97.7%)
1 Region	10 (1.2%)	8 (1.0%)	6 (0.7%)
2 Regions	3 (0.4%)	6 (0.7%)	7 (0.9%)
3 Regions	3 (0.4%)	1 (0.1%)	3 (0.4%)
4 Regions	3 (0.4%)	3 (0.4%)	3 (0.4%)
5 Regions	5 (0.6%)	3 (0.4%)	0 (0.0%)

## Discussion

Low back pain is common in a large, working occupational cohort, with 20.8% having LBP on the day of enrollment and 44.0% having had pain in the prior month. A total of 63.5% reported having had LBP lasting at least one day in his or her lifetime. The majority of point prevalent and 1-month period prevalent pain was reported in the immediate paraspinal area. Similar amounts of pain were reported in the right and left sides, but few reported gluteal pain in contrast with clinical experiences. Pain ratings varied widely, with a considerable number of participants having high pain ratings despite being apparently functional at work. Tingling and numbness in the lower extremities were relatively rare. Anecdotally, these results suggest many workers “live with” LBP and continue working, perhaps until pain either worsens or is intolerable.

As the mean age of the population at enrollment was 38 years, it is likely a portion of workers reporting no LBP history had not lived long enough to develop LBP while others may have forgotten prior pain, thus similar to other studies the lifetime cumulative prevalence estimate may substantially understate the true lifetime risk[2, 5, 8–13]. While these prevalence measures are comparable with results from prior publications, to our knowledge, this is the first detailed reporting of pain ratings and anatomic locations[2, 5, 8–13].

Pain ratings in an at-work population were considerably higher than expected, with 116 providing one-month LBP ratings classified as low, 170 medium and 78 with high pain ratings. Pain ratings on the day of enrollment ranged widely with 58 rating low, and 75 providing LBP ratings of medium. Because all participants were working at the time of data collection, we were surprised that an extraordinary 39 of 828 (4.7% of total participants, 22.7% of those with point prevalent pain) were also reporting high pain ratings at that time. These high pain ratings are generally synonymous with “severe” pain and which many have considered incompatible with productive work. Of those working with high pain ratings, only one was on light duty, and none of them saw a healthcare provider before the next monthly follow up.

A total of 114 reported pain ratings on the day of enrollment classified as medium or high. Many randomized trials investigating opioid treatment for LBP have had minimum pain rating inclusion criteria[25]. Trials for treatment of acute or subacute LBP have utilized inclusion criteria of at least 4/10 or 5/10 pain ratings[40–43]. Trials assessing chronic pain had similar requirements[44–50]. Similar minimum pain requirements are used in trials investigating muscle relaxants for acute and sub-acute LBP treatment[51, 52]. In this study, 114 participants (18.8%) had point prevalence pain meeting the criterion of ≥4/10 for opioid treatment on the day of enrollment and 248 (30.0%) met that criterion over the month prior to enrollment. As these participants were functional at work, these results raise concerns regarding the use of pain ratings as either a sole or primary treatment criterion[53–55].

For this study, pain ratings of 7 to 10 out of 10 were considered high, or “severe”. These findings raise concerns about the efforts to record pain ratings in all patients[12, 56–59]. Instead, a focus on function has been increasingly advocated[25, 60–62]. As these results are from a large working population yet with considerable pain ratings, these data appear to provide indirect support for efforts to assess pain via a function-based approach.

There are few studies which have reported on the geographic distribution of LBP, with most reporting pain between the ribs and buttocks. This study found a high proportion of patients (278 of 828, 33.6%) with paraspinal pain in the past month. However, in a clinical setting patients frequently present with gluteal pain[63, 64]. Thus, the small proportions with point or 1-month period prevalence of gluteal pain were unexpected. Additionally, some believe that radiating pain, including into the gluteal areas signifies mild radiculopathy, however, the frequencies of sciatica in this study add to other evidence that suggest gluteal pain may be merely referred pain[25]. The utilization of a standardized, a priori back diagram instead of relying only on a pain picture may partially explain the lower prevalence of gluteal pain.

Additionally, there were a surprising number of participants who report pain in more than one low back area. 68/172 (39.5%) reported point prevalence pain in more than one low back area, as did 157/364 (43.1%) of those reporting 1-month period prevalence. Clinical anecdotes, which are all that are available in the literature, describe most patients as having relatively focused LBP, with few reporting wide-spread, diffuse LBP. These *a priori* divisions of LBP are based upon clinical experience of the researchers, and were selected to evaluate suspected differentiations of pain location. While there are multiple reports on widespread musculoskeletal pain in different body areas, the differentiation between focal point LBP and more widespread LBP has never been reported in the literature. These widespread pain areas, with many participants reporting pain in 3, 4 and all 5 areas, contradicts the concept that LBP manifests with one specific point of pain, as may be seen clinically. It is unknown as to the relationships, if any, between specific location(s) of LBP and either causal or prognostic factors. Evaluation of the relative importance of LBP location and associated factors may be possible. Also, there may be multiple etiological factors related to pain in different or multiple back locations, and/or psychosocial factors and assessment of these potential relationships are areas of further research.

Not surprisingly there are fewer prior reports of point prevalence compared to one-month period prevalence pain. Point prevalence and the corresponding LBP ratings are generally lower. However, this study found similarities between point prevalence and one month period prevalence pain in the range of pain ratings and the distribution of anatomical locations. We had expected the distribution of pain ratings to shift towards higher pain ratings when workers were asked to recall one-month LBP period prevalence instead of LBP point prevalence. Instead, we were surprised to find relatively little change in the distribution.

Strengths of this study include the large sample size, numerous employment settings involvement of employers in four diverse US states, complete data capture utilizing trained healthcare providers, a computerized structured interview and use of a body diagram to collect detailed information including pain locations and pain ratings. While younger and healthier than the general population, this dataset is similar to other occupational cohorts in age and proportion of chronic diseases such as diabetes mellitus, thyroid disease, smoking, body mass index[27–32, 65–70]. While we are unable to directly test potential differences between responders and non-responders, the comparability of the dataset to national expectations suggests these results are likely generalizable to other employed populations especially of manual material handling workers.

Weaknesses include reliance on primarily manufacturing and warehousing employee groups that may limit generalizability. Also, as there are no objective measures of LBP, this study relies on self-reported pain ratings. This study is subject to recall biases. Although there were no known labor strife issues to provoke such issues. Some underestimation due to workers absent with LBP on the day of enrollment is possible, although we enrolled at sites on multiple dates and intentionally sought to enroll those not present at the prior enrollments, thus reducing this potential source of selection bias. Additionally, research suggests that recalled pain ratings are relatively stable over a week, and it may be reasonable to expect that recall of pain ratings are similarly stable at 1 month[71].

## Conclusions

There are variations in the pain ratings of both point prevalence and 1-month period prevalence measures of LBP within this large population, with smaller variation in pain ratings within different locations. These variations have not been described in the literature. These analyses demonstrate that there is variation of location and intensity of pain, which have commonly been classified in epidemiological studies evaluating potential causal factors as one binary LBP case by researchers and clinicians, regardless of pain location or intensity between the 12^th^ rib and the gluteal fold. By aggregating these different locations of pain or different intensities of pain into one binary classification of LBP, researchers and clinicians may be losing important information about individuals that may be useful in prevention or treatment of LBP. Further research is needed to identify associations between variations in both intensity and location and different etiological factors related to LBP. Additional analyses of LBP within this cohort, including intensity and specific location of incident LBP cases which demonstrate temporality, may yield further insight regarding relationships between pain location or intensity and other factors such as psychosocial problems, co-morbid chronic diseases, or job physical factors.

## Authors’ original submitted files for images

Below are the links to the authors’ original submitted files for images. Authors’ original file for figure 1 Authors’ original file for figure 2

## Abbreviation

LBP: Low back pain.
