# Supplementary figures and images for: Prevalence of low back pain by anatomic location and intensity in an occupational population
Source: BMC Musculoskelet Disord. 2014 Aug 21;15:283. doi: 10.1186/1471-2474-15-283 (PMC4153910; doi:10.1186/1471-2474-15-283)

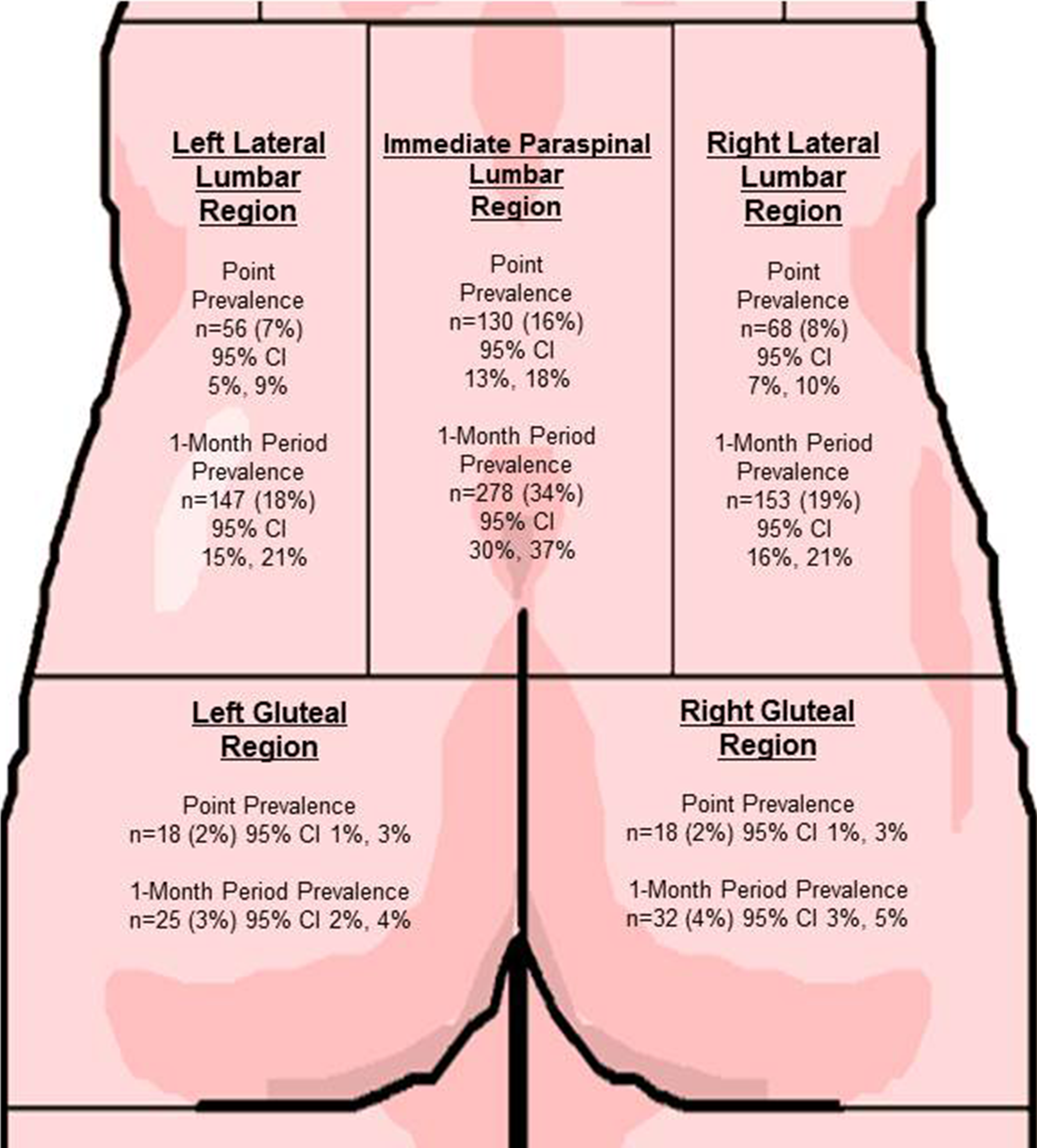

Supplement: Supplementary file 1 — Authors’ original file for figure 1 [file 12891_2014_2229_MOESM1_ESM.tif]

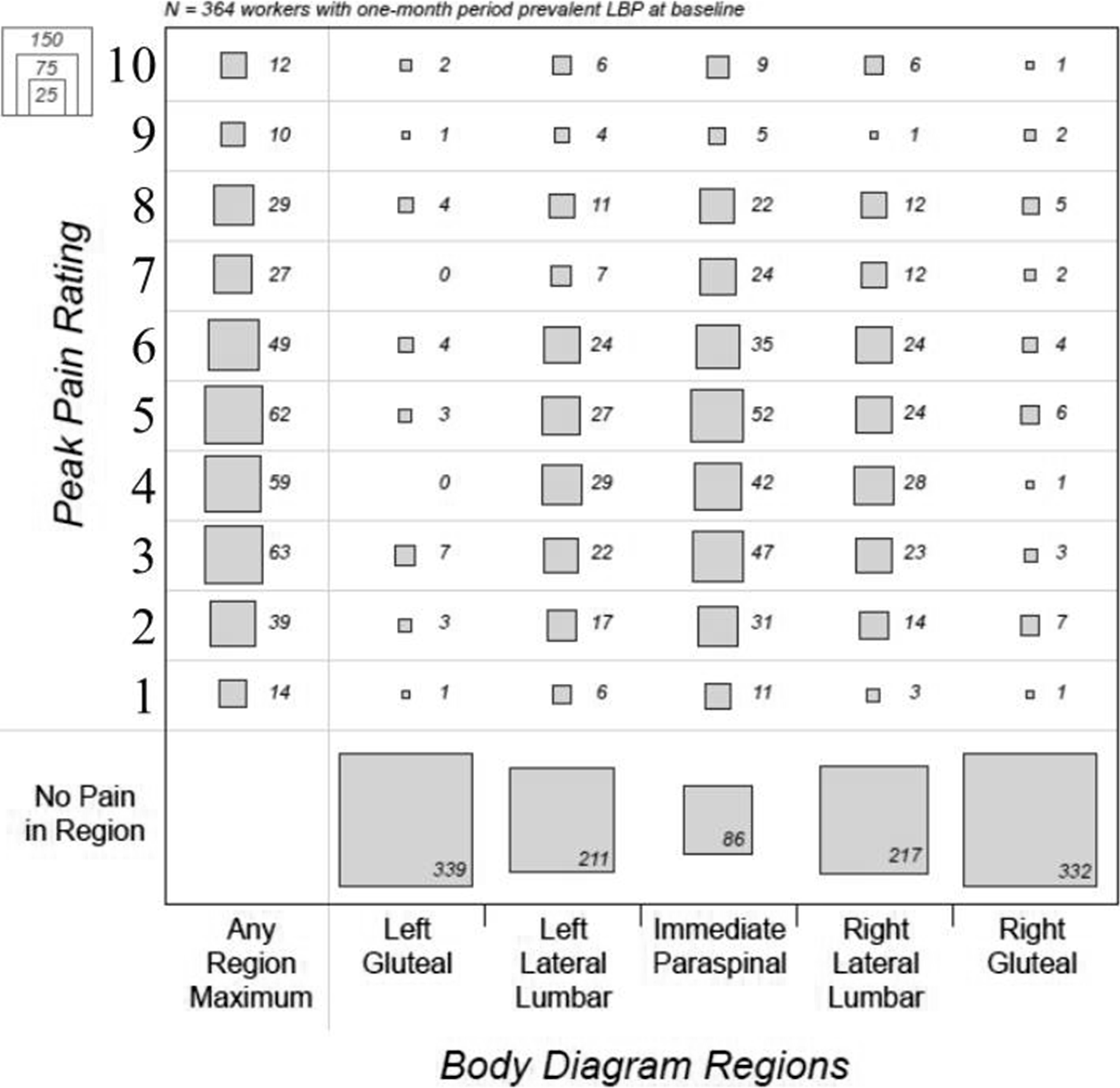

Supplement: Supplementary file 2 — Authors’ original file for figure 2 [file 12891_2014_2229_MOESM2_ESM.tif]
